# Luteolin-7-O-β-d-Glucuronide Attenuated Cerebral Ischemia/Reperfusion Injury: Involvement of the Blood–Brain Barrier

**DOI:** 10.3390/biomedicines12061366

**Published:** 2024-06-19

**Authors:** Xing Fan, Jintao Song, Shuting Zhang, Lihui Lu, Fang Lin, Yu Chen, Shichang Li, Xinxin Jin, Fang Wang

**Affiliations:** 1 School of Life Science and Biopharmaceutics, Shenyang Pharmaceutical University, Shenyang 110016, China; fanxingspu@163.com (X.F.); lulihui@cms.net.cn (L.L.); linfang66889@sina.com (F.L.); 18333323623@163.com (S.L.); 2 School of Functional Food and Wine, Shenyang Pharmaceutical University, Shenyang 110016, China; 18896656392@163.com (J.S.); zstzwz-3@163.com (S.Z.); chenyu2769@163.com (Y.C.); 3 Experimental Teaching Center of Pharmacology, Shenyang Pharmaceutical University, Shenyang 110016, China; lingyuan-009@163.com

**Keywords:** luteolin-7-O-β-D-glucuronide, middle cerebral artery occlusion, cerebral ischemia/reperfusion, blood–brain barrier, inflammation

## Abstract

Ischemic stroke is a common cerebrovascular disease with high mortality, high morbidity, and high disability. Cerebral ischemia/reperfusion injury seriously affects the quality of life of patients. Luteolin-7-O-β-d-glucuronide (LGU) is a major active flavonoid compound extracted from *Ixeris sonchifolia (Bge.)* Hance, a Chinese medicinal herb mainly used for the treatment of coronary heart disease, angina pectoris, cerebral infarction, etc. In the present study, the protective effect of LGU on cerebral ischemia/reperfusion injury was investigated in an oxygen–glucose deprivation/reoxygenation (OGD/R) neuronal model and a transient middle cerebral artery occlusion (tMCAO) rat model. In in vitro experiments, LGU was found to improve the OGD/R-induced decrease in neuronal viability effectively by the MTT assay. In in vivo experiments, neurological deficit scores, infarction volume rates, and brain water content rates were improved after a single intravenous administration of LGU. These findings suggest that LGU has significant protective effects on cerebral ischemia/reperfusion injury in vitro and in vivo. To further explore the potential mechanism of LGU on cerebral ischemia/reperfusion injury, we performed a series of tests. The results showed that a single administration of LGU decreased the content of EB and S100B and ameliorated the abnormal expression of tight junction proteins ZO-1 and occludin and metalloproteinase MMP-9 in the ischemic cerebral cortex of the tMCAO 24-h injury model. In addition, LGU also improved the tight junction structure between endothelial cells and the degree of basement membrane degradation and reduced the content of TNF-α and IL-1β in the brain tissue. Thereby, LGU attenuated cerebral ischemia/reperfusion injury by improving the permeability of the blood–brain barrier. The present study provides new insights into the therapeutic potential of LGU in cerebral ischemia.

## 1. Introduction

Globally, stroke is a significant cause of death and permanent disability, with ischemic stroke accounting for over 87% of cases. The crucial aspect of treatment involves promptly restoring normal blood circulation and reperfusing ischemic brain tissue [1,2,3]. However, its significant clinical limitations and narrow treatment window heighten the risk of intracerebral hemorrhage in the brain [4]. A series of cellular, biochemical, and metabolic consequences will occur whether it is during ischemia or reperfusion, including the production of intracellular ROS and RNS, intracellular Ca^2+^ overload [5], glutamate neurotoxicity, inflammation, and cell apoptosis. For brain tissue, these pathological reactions eventually cause permanent damage [6]. This phenomenon is called cerebral ischemia/reperfusion injury, which is not ischemia itself but the reperfusion of ischemic tissue, leading to the further aggravation of ischemic brain dysfunction.

Cerebral ischemia/reperfusion injury is a rapid and complex physiological and pathological process, including primary injury during ischemia and secondary injury during reperfusion [7]. The injury mechanisms mainly include excitatory amino acid toxicity, intracellular Ca^2+^ overload, free radical damage, immune inflammatory response, apoptosis, blood–brain barrier (BBB) destruction, etc. [8,9,10], which can cause intracranial hemorrhage and brain edema and other adverse reactions.

A unique microenvironment essential for normal brain function and homeostasis is established by the highly selective structural and functional barrier known as the BBB, which separates the blood from the central nervous system [11]. The broad blood–brain barrier consists of three parts [12] including the blood–brain barrier, the blood–cerebrospinal fluid barrier, and the cerebrospinal fluid–brain barrier. The damage mechanism of BBB permeability change mainly includes [13,14] the following: affecting the expression and distribution of tight connection-related proteins; activating matrix metalloproteinases; releasing a large number of inflammatory mediators; increasing the expression of S100 calcium-binding protein B in the blood; and increasing the expression of aquaporin in astrocytes.

Luteolin-7-O-β-D-glucuronide (LGU) is the main active monomer compound with a flavonoid structure and is one of the main active ingredients with the highest content in *Ixeris sonchifolia (Bge.)* Hance. After years of development, injections made with extract of *Ixeris sonchifolia (Bge.)* Hance have been clinically used to treat angina pectoris, coronary heart disease, acute cerebral infarction, diabetes, and other diseases [15]. Our previous studies found that LGU has a protective effect on the OGD neuron model, and its mechanism is related to inhibiting calcium overload, improving mitochondrial function, and inhibiting necroptosis in vitro. LGU also has a significant protective effect on the pMCAO rat model, and its mechanism is related to significantly improving energy metabolism disorder, mitochondrial function, and reducing inflammatory response injury. However, the underlying mechanism by which LGU exerts its protective effects remains unclear, particularly in regard to its impact on BBB permeability. In this study, we aim to investigate the potential role of LGU in regulating BBB permeability during cerebral ischemia/reperfusion injury using an OGD/R neuronal model and a tMCAO rat model. By filling this gap in knowledge, our findings may contribute to the development of novel therapeutic approaches for the treatment of cerebral ischemia/reperfusion injury.

## 2. Materials and Methods

### 2.1. Materials

S100B kits (H258) were purchased from Nanjing Jiancheng Biological Engineering Institute. Anti-MMP-9 antibodies (ab76003) and anti-occludin antibodies (ab216327) were purchased from Abcam (Cambridge, UK). Anti-ZO-1 antibodies (WL03419) were purchased from Wanlei Biotechnology Co., Ltd. (Shenyang, China).

### 2.2. Experimental Animals

Neonatal SD rats (within 24 h) and adult SD male rats, SPF grade, weighing 260~280 g, were provided by Liaoning Changsheng Biotechnology Co., Ltd. (Benxi, China), License number: SCXK (Liao) 2015-0001. The rats had access to food and water ad libitum and were allowed to adapt to the environment for four days prior to the experiments. Experiments were performed at a room temperature of 22–24 °C and a room humidity of 40–60%. The experimental protocol used in this study was approved by the Animal Ethics Committee of Shenyang Pharmaceutical University. The routine experiments were performed in accordance with the “Regulations on the Management of Laboratory Animals” issued by the National Science and Technology Commission [16]. Neonatal Sprague-Dawley rats were used for OGD/R experiments to culture primary cortical neurons. The rats were randomly divided into the sham operation group, model group, and LGU treatment group (0.24, 0.72, 2.16 mg/kg). Adult male Sprague-Dawley rats were used for MCAO experiments.

### 2.3. Primary Culture of Cerebral Cortical Neurons

After being dissected from the brain of neonatal SD rats, the cortex was placed in a DMEM/F12 medium precooled at 4 °C containing 10% fetal bovine serum. About 5 times the tissue volume of 0.25% Trypsin–EDTA was added to the tissue fragments. After mixing, it was inhaled into the sterilized centrifugal glass test tube and placed in a 37 °C water bath for accurate digestion for 15 min. The supernatant was sucked up with a rubber head dropper, and then the planting culture medium was added. A double-layer 200-mesh cell filter was used to obtain a nerve cell suspension, which was planted in a 48-well plate precoated with 0.01% polylysine, 200 μL/well, and placed in 37 °C, 5% CO_2_ incubator. After 4 h of attachment, the culture medium was replaced with Neurobasal medium preheated at 37 °C (containing 2% B27 supplement and 1% double antibody), and then the culture medium was replaced half every 72 h. Neuronal cells could be cultured until day 7 for experiments [17].

### 2.4. Experimental Protocol for the Protective Effect of LGU on OGD/R Neurons

The solvent was Neurobasal Medium + 2% B27 Supplement + 1% double antibody culture medium. Primary neurons were cultured in 48-well plates. The cells in the control group were cultured in a 5% CO_2_ incubator at 37 °C. The cells in the model (OGD/R) group and the LGU groups were incubated in a conventional feeding solution, and then preheated sugar-free DMEM was added, and the cells were placed in a hypoxic chamber (95% N_2_, 5% CO_2_) for 12 h and 16 h OGD. After the end of OGD, the LGU working solution (0.37, 1.2, 3.7, 11, 33, 100 μM) prepared with Neurobasal Medium, 2% B27 Supplement and 1% double-antibody culture medium was added to the culture plate. MTT experiments were performed after reperfusion for 36 h, 48 h, and 72 h [18].

### 2.5. tMCAO Model

The tMCAO model was prepared according to the method of Zea Longa [19]. The rats were fasted for 12 h before operation. The rats were anesthetized with inhaled isoflurane, fixed in the supine position, shaved at the neck, and cut 2–3 cm in the median line of the neck. The fascia muscles were bluntly separated, and the carotid triangle was fully exposed. Then, the right common carotid artery (CCA), external carotid artery (ECA), and internal carotid artery (ICA) were bluntly separated. The 5-0 suture was used to prepare the wire at the distal end of the CCA and separate other small branches. At the same time, the distal and proximal ends of the ECA were ligated, and the electrocoagulator was coagulated in the middle of both ends to keep the root of the ECA as long as possible. The rats were divided into a sham operation group, tMCAO 12 h, tMCAO 24 h, and tMCAO 48 h. After OGD2h, reperfusion was performed for 12 h, 24 h, and 48 h, respectively. The neurological function score, TTC staining, and brain water content were evaluated.

### 2.6. Neurological Function Score

The neurobehavioral changes in rats in each group were observed at different ischemic time points, according to the 5-level scoring system reported by Longa et al. [19].

### 2.7. TTC Staining

Coronal brain slices were cut from the frontal pole. Then, the brain slices were placed in a six-well plate coated with tin foil and containing 1% TTC solution, incubated at 37 °C for 15 min. After TTC staining, the brain slices were fixed in a 4% paraformaldehyde solution in the dark and photographed. Image Pro Plus 6.0 was used to analyze the area of cerebral infarction, and the infarct volume ratio was calculated according to the following formula [20]:

$$Infarct\;volume\;ratio\;\%=\frac{Infarction\;size}{Ipsilateral\;cerebral\;hemisphere\;volume}\times 100\%$$

### 2.8. Brain Water Content

The whole brain tissue weight (brain wet weight) of each group was recorded by an analytical balance (AL104-IC). After 1% TTC staining, the brain slices were dried to constant weight in an oven at 37 °C, and the weight (brain dry weight) was recorded. The brain water content was calculated according to the following formula [21]:

$$Brain\;water\;contents\;\%=\frac{Brain\;wet\;weight\;-\;Brain\;dry\;weight}{Brain\;wet\;weight}\times 100\%$$

### 2.9. Experimental Protocol for the Protective Effect of LGU on tMCAO Rats

The tMCAO model was evaluated before administration. Except for the sham operation group, the tMCAO model rats should have left anterior limb dysfunction [22]. The LGU treatment group (0.24, 0.72, and 2.16 mg/kg) and the ED-positive drug group (5 mg/kg) were given the corresponding drugs by tail vein immediately after reperfusion. The sham operation group and the model group were given an equal volume of solvent at the same time point. Neurological function score [23], cerebral infarction volume ratio and brain water content were measured in each group 22 h after reperfusion.

### 2.10. Evans Blue

Evans blue (2% EB, 4 mL/kg) was injected into the tail vein of each group 20 h after reperfusion. After the Evans blue dye was fully circulated in SD rats, 3.5% chloral hydrate was injected intraperitoneally at the corresponding time point 2 h later. Then, 0.9% normal saline was quickly pushed to rinse the blood in the rat until the Evans blue dye in the blood circulation was perfused clean (clear liquid flowed out of the right atrial appendage incision), and the brain was quickly decapitated to remove the cerebellum and brainstem.

The wet weight of the ischemia cerebral hemisphere was weighed, and then the injured cerebral tissue was fully ground in 3 mL of a formamide aqueous solution, incubated in 60 °C water bath for 48 h, and centrifuged at 5000 r/min at 4 °C for 15 min, after which the supernatant was taken. Then, 200 μL of the supernatant was added to 96-well plates and the optical density was measured at 630 nm. The content of EB in the sample was calculated by comparing the standard curve.

$$EB\;content\;in\;brain\;tissue\;(µg/g)\;=\;\frac{EB\;content\;of\;sample\;to\;be\;tested\;(µg/mL)\;\times \;Formamide\;amount\;(mL)}{Brain\;tissue\;wet\;weight\;(g)}$$

### 2.11. Detection of S100B Content in Rat Serum by the Elisa Kit

The cerebral tissue of the ischemic hemispheres of rats was prepared into 8% tissue homogenate, centrifuged at 4 °C, 3000 r/min, for 15 min, and then the supernatant was taken and stored in a refrigerator at −80 °C.

According to the operation instructions of the kit, the absorbance value was measured at the wavelength of 562 nm, and the protein concentration of the sample was calculated according to the OD value of the sample and the standard curve.

The content of S100B in serum was determined by the competition method, and the absorbance (OD value) was detected at a 450 nm wavelength. The concentration of S100B in the sample was calculated by the standard curve, and the OD value was negatively correlated with the concentration of antigen in the sample. All experimental procedures were conducted in strict accordance with the Nanjing-built kit operation.

### 2.12. Electron Microscope Study

After 24 h of ischemia, 3 rats were randomly selected from each group. After anesthesia, 1 mm × 1 mm rice grain tissue blocks were taken from the frontal cortex of the frontal lobe and fixed with osmic acid. The samples were then dehydrated by gradient ethanol–acetone step by step and then embedded in Spurr resin. The copper mesh with tissue sections was immersed in a uranium acetate dye solution and stained for 30 min away from light. Then, a lead citrate stain solution was added to the Petri dish, and the dyeing was continued for 10 min. H-7650 transmission electron microscopy (Hitachi, Chiyoda, Tokyo) was used to analyze the slices at 40 nm thickness.

### 2.13. Detection of the Expression of Blood–Brain Barrier-Related Proteins by Western Blot

The protein samples were loaded on 10% acrylamide gel for electrophoresis. They were occluded in 5% skim milk for 1 h, then gently shaken with the primary antibody on a shaker and incubated at 4 °C overnight (primary antibody concentration: ZO-1 1:1000, MMP-9 1:500, occludin 1:1000). B-actin was used as the internal parameter to ensure equal loading. After washing with TBST buffer, the samples were incubated with an enzyme-labeled secondary antibody. The secondary antibody was left at room temperature for 1 h. Finally, a PVDF reagent was used to detect the membrane, and a gel image analysis imaging system was used for the scanning analysis. The results were represented by the relative expression of the target protein using Image J 1.46r image processing software.

$$The\;relative\;expression\;of\;target\;protein\;=\;\frac{Objective\;protein\;integral\;optical\;density\;value}{Integral\;optical\;density\;value}$$

### 2.14. Detection of the Changes in TNF-α and IL-1β in Ischemic Brain Tissue of Rats Using the Elisa Kit

The content of TNF-α and IL-1β in the ischemic brain tissue was detected by the double antibody sandwich method. The OD value was detected at a 450 nm wavelength. The concentration of TNF-α and IL-1β in the sample was calculated by the standard curve. The OD value was positively correlated with the concentration of the antigen in the sample. The experimental steps were operated strictly according to the enzyme immunoassay kit.

### 2.15. Data Analysis

Image Pro-Plus6 was used to calculate the cerebral infarction volume ratio. Data were expressed as mean ± S.E.M. Statistical analyses were performed using IBM SPSS (Version 21.0, Armonk, NY, USA). One-way analysis of variance (ANOVA) was used to assess the differences in all observed indicators among the different groups. The Dunnett test was used for multiple comparisons. Statistical significance was set at *p* < 0.05.

## 3. Results

### 3.1. Establishment of the OGD/R Model in Rat Cerebral Cortical Neurons

**Figure 1:**
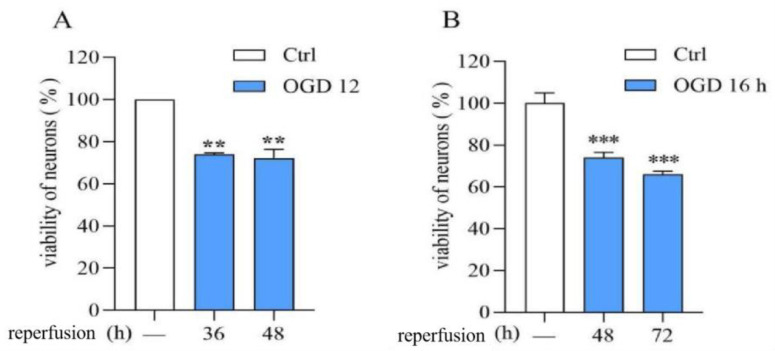
Establishment of the OGD/R model in rat cerebral cortical neurons. (**A**): OGD/R:12 h/36 and 48 h. (**B**): OGD/R: 16 h/48 and 72 h. Data are expressed as mean ± S.E.M. (n = 3). ** *p* < 0.01 *** *p* < 0.000 vs. the ctrl group.

Compared with normal neuronal viability, OGD (12 h or 16 h)/R (36, 48, or 72 h) significantly reduced neuronal viability. The OGD/R injury model was successfully established (Figure 1).

### 3.2. Protective Effect of LGU on the OGD/R-Induced Decrease in Cell Viability in Rat Primary Cortical Neurons

**Figure 2:**
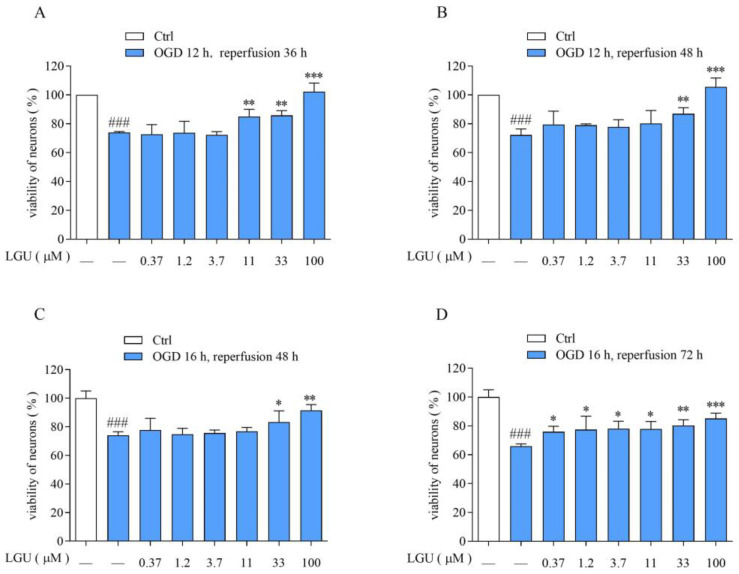
Protective effect of LGU on the OGD/R-induced decrease in cell viability in rat primary cortical neurons. (**A**): OGD/R:12 h/36 h. (**B**): OGD/R: 12 h/48 h. (**C**): OGD/R:16 h/48 h. (**D**): OGD/R: 16 h/72 h.Data are expressed as mean ± S.E.M. (n = 3). ### *p* < 0.001 vs. the ctrl group, * *p* < 0.05, ** *p* < 0.01, *** *p* < 0.000 vs. OGD/R group.

The neuronal viability in OGD/R was significantly lower than that of normal neurons. LGU (0.37–100 μM) effectively attenuated the OGD/R-induced decrease in cell viability with different degrees (Figure 2).

### 3.3. Establishment of the tMCAO Rat Model

**Figure 3:**
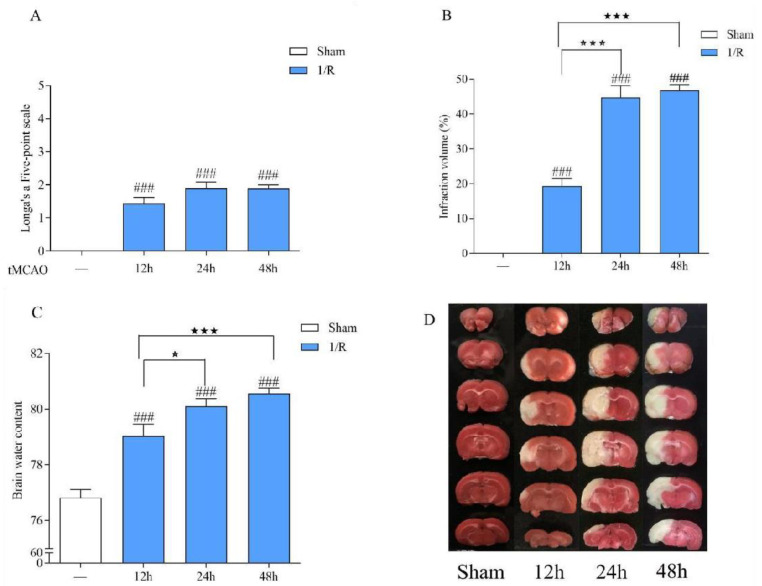
Establishment of the tMCAO rat model. (**A**): Neurological function score. (**B**): Cerebral infarct volume. (**C**): Brain water content. (**D**): Representative cerebral infarction image. Data are expressed as the mean ± S.E.M. n = 9–10. ### *p* < 0.001, compared with the sham group; ★ *p* < 0.05, ★★★ *p* < 0.001 compared with the tMCAO 12 h group.

The experimental results are shown in Figure 3. Compared with the sham operation group, the neurological function score in the tMCAO model rat was significantly increased after 12 h, 24 h, and 48 h of ischemia/reperfusion, the volume ratio of cerebral infarction and the brain water content were increased, suggesting that rats had obvious neurological dysfunction, cerebral ischemia, and cerebral edema and that the model was successfully prepared. This shows that the model damage was more significant and stable after 24 h of ischemia/reperfusion. Therefore, tMCAO 24 h was selected as the time point to study the efficacy of LGU.

### 3.4. Protective Effect of LGU on Ischemic Brain Injury in tMCAO Rats

**Figure 4:**
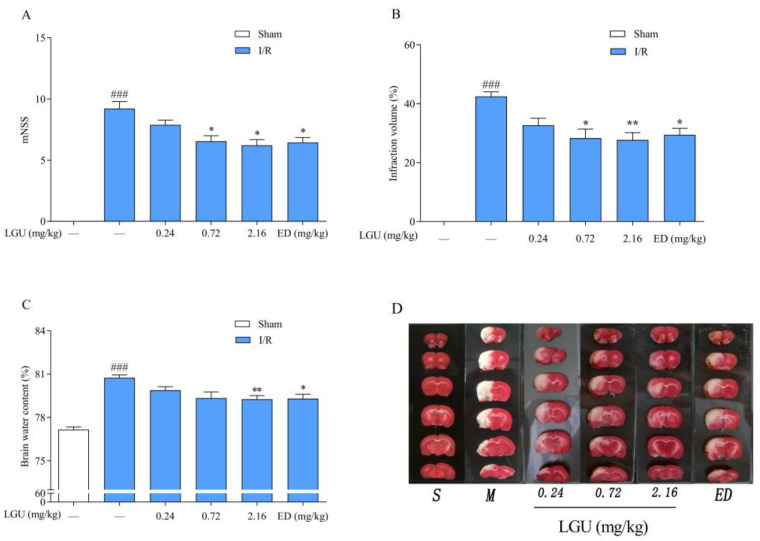
Protective effect of LGU on ischemic brain injury in tMCAO rats. (**A**): Neurological function score. (**B**): Cerebral infarct volume. (**C**): Brain water content. (**D**): Representative cerebral infarction image. Data are expressed as the mean ± S.E.M. n = 7–9. ### *p* < 0.001, compared with the sham group; * *p* < 0.05, ** *p* < 0.01, compared with the tMCAO model group.

The experimental results are shown in Figure 4. The volume of cerebral infarction was observed by 1%TTC staining. The normal brain tissue was red by TTC staining, while the infarct tissue was white. Compared with the sham group, the neurological function score, cerebral infarction volume ratio, and brain water content of the tMCAO model group were significantly increased. Compared with the tMCAO model group, LGU (0.72, 2.16 mg/kg) significantly reduced the abnormal increase in the neurological function score and cerebral infarction volume ratio, and LGU (2.16 mg/kg) also improved the change in the brain water content in the tMCAO model group. The results showed that LGU had a significant protective effect on the tMCAO rat model in a dose-dependent manner.

### 3.5. Effect of LGU on the Permeability of Evans Blue in tMCAO Rats

**Figure 5:**
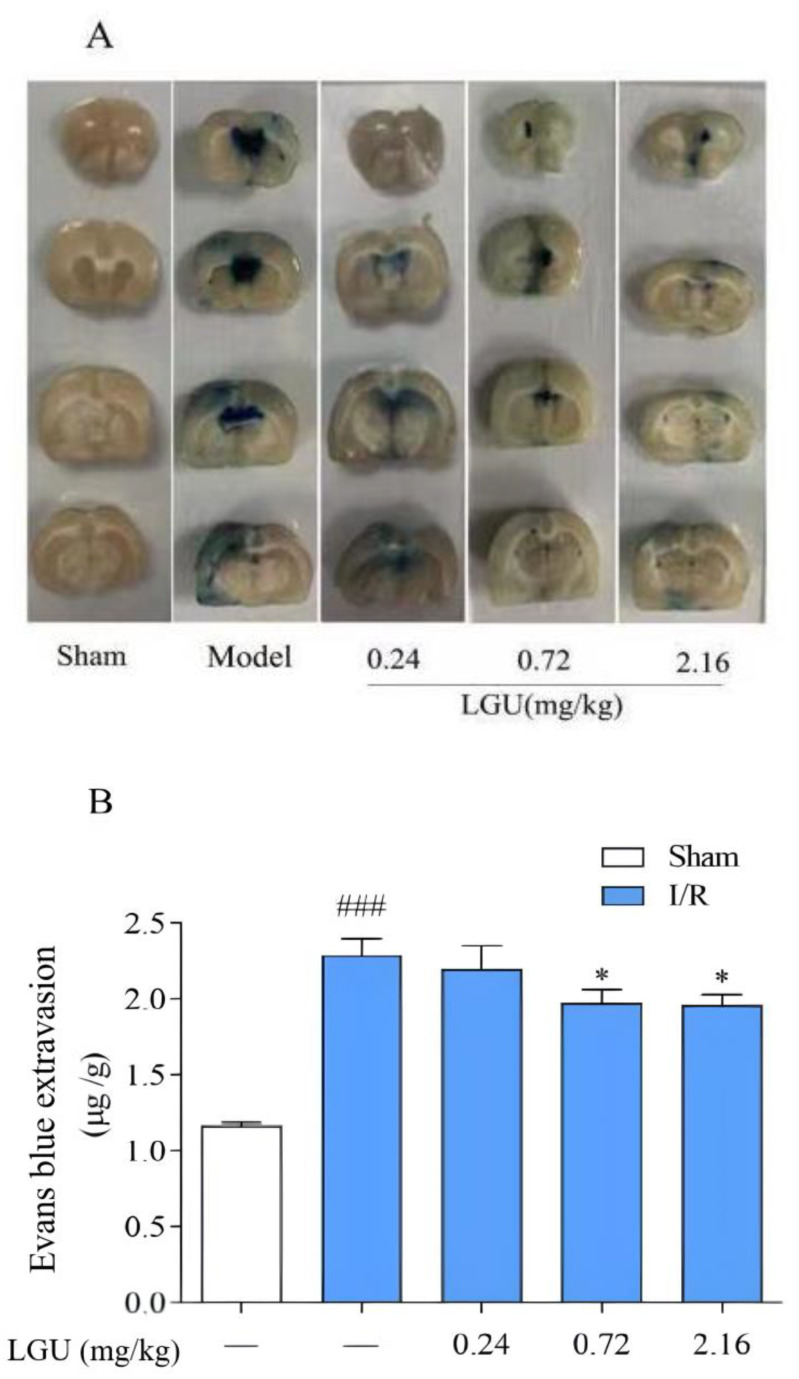
Effect of LGU on the permeability of Evans blue in tMCAO rats. (**A**): Representative images of EB leakage in the cerebral cortex. (**B**): Quantitative analysis of EB leakage. Data are expressed as the mean ± S.E.M.; n = 6–7. ### *p* < 0.001, compared with the sham group; * *p* < 0.05, compared with the model group.

EB was injected into the tail veins two hours before sampling, and the whole body of the rats became blue rapidly. After 24 h of ischemia–reperfusion, the integrity of the blood–brain barrier was evaluated by detecting the amount of EB exudation in the brain tissue. The normal brain tissue was white, and the blue area was EB exudation. Compared with the sham operation group, the ischemic side of the model group showed obvious leakage (Figure 5). After a single tail vein administration of different concentrations of LGU, compared with the model group, the EB penetration in the LGU group (0.72, 2.16 mg/kg) was significantly reduced.

### 3.6. Effect of LGU on the Content of S100B in the Serum of tMCAO Rats

**Figure 6:**
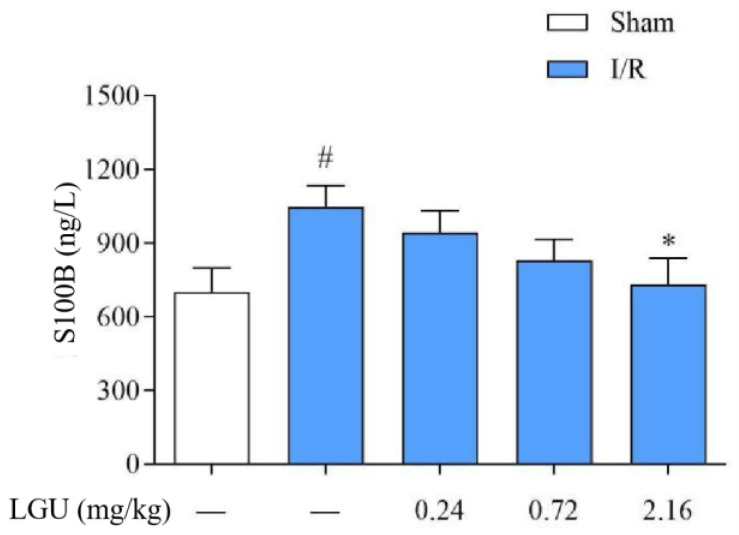
Effect of LGU on the content of serum S100B in tMCAO rats. Data are expressed as mean ± S.E.M. n = 7. # *p* < 0.05, compared with the sham group; * *p* < 0.05, compared with the model group.

The content of S100B in the serum of rats from the model group was significantly increased compared with the sham group, as indicated in Figure 6. Following a single tail vein injection of LGU (2.16 mg/kg), there was a notable reduction in the S100B content in the serum of rats with cerebral ischemia.

### 3.7. Effects of LGU on the Ultrastructural Changes in Neurons in tMCAO Rats

**Figure 7:**
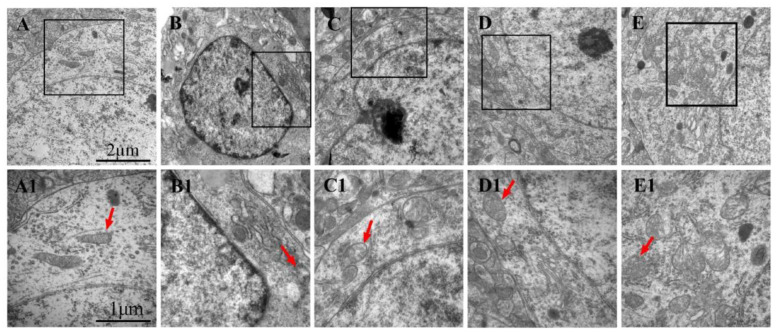
Effects of LGU on the ultrastructural changes in neurons in tMCAO rats: (**A**–**E**): 20,000× and (**A1**–**E1**): 40,000×. Mitochondria (red arrowhead) were observed. (**A**,**A1**): Sham; (**B**,**B1**): model; (**C**,**C1**): LGU (0.24 mg/kg); (**D**,**D1**): LGU (0.72 mg/kg); and (**E**,**E1**): LGU (2.16 mg/kg).

The ultrastructure of neurons in the sham operation group (Figure 7A,A1) was normal. Compared with the sham operation group, the ultrastructure of neurons in the model group was significantly damaged, and the chromatin distribution in the nucleus was uneven. The mitochondria in the cytoplasm were reduced, and the mitochondria with rich internal cristae were significantly reduced. A few mitochondria were swollen, and the intercristal cavity was enlarged and vacuolated. Compared with the model control group, the LGU (0.24 mg/kg) group showed an incoherent nuclear membrane structure of neurons and mild swelling of mitochondria in the cytoplasm, but the structure of the rough endoplasmic reticulum and ribosomes was clear. In the LGU (0.72 mg/kg) (2.16 mg/kg) group, the structure of the nuclear membrane was complete, the number of mitochondria in the cytoplasm was increased, the internal ridge was clear and neat, the mitochondria with high transparency were obviously increased, and the internal ridge was clear and neat with high transparency.

### 3.8. Effects of LGU on the Ultrastructural Changes in the Blood–Brain Barrier in tMCAO Rats

**Figure 8:**
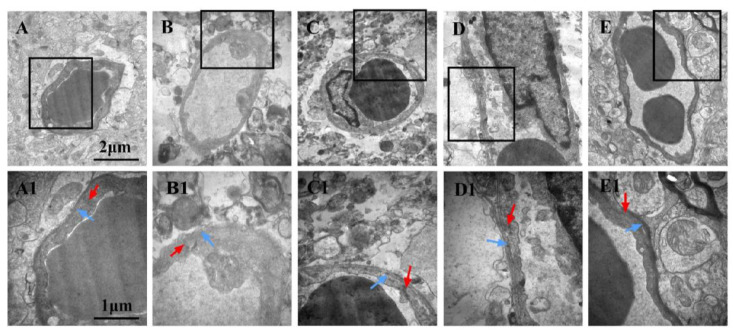
Effects of LGU on the ultrastructural changes in the blood–brain barrier in tMCAO rats. The photomicrographs in (**A1**), (**B1**), (**C1**), and (**D1**) exhibit higher magnification images of the areas shown in frames in (**A**), (**B**), (**C**), and (**D**), respectively. The basement membrane (blue arrowhead) and tight junction (red arrowhead) were observed. (**A**,**A1**): Sham; (**B**,**B1**): model; (**C**,**C1**): LGU (0.24 mg/kg); (**D**,**D1**): LGU (0.72 mg/kg); (**E**,**E1**): LGU (2.16 mg/kg); (**A**–**E**): 20,000×; and (**A1**–**E1**): 40,000×.

In the sham operation group (Figure 8A,A1), the cerebral vascular structure of the rats was normal. Compared with the sham operation group, the perivascular space of the model group (Figure 8B,B1) became wider, the tissue was sparse, and there was obvious edema. There was necrosis and large blanks in the perivascular tissue, and vacuolar changes appeared. Under high magnification, the tight junction between vascular endothelial cells began to become blurred, the thickness of the vascular basement membrane was uneven, and dissolution occurred. The glial cells around the basement membrane were dissolved and disappeared, and the mitochondria in the cytoplasm were swollen. Compared with the model control group, the LGU (0.24 mg/kg) group (Figure 8C,C1) had obvious local edema around the blood vessels, and there were still large blanks around the blood vessels. The vascular morphology was irregular, the thickness of the vascular basement membrane was uneven, and the integrity was destroyed under high magnification. In the LGU (0.72 mg/kg) group (Figure 8D,D1), the degree of perivascular edema was reduced, the capillary cavity surface was rough, and nodular enlargement was formed locally. In the LGU (2.16 mg/kg) group (Figure 8E,E1), perivascular edema was slight, the close connection between vascular endothelial cells was not broken, the basal layer was in close contact with endothelial cells, the basal membrane was intact, and the ultrastructure of the blood–brain barrier was significantly improved.

### 3.9. Effects of LGU on the Expression of Tight Junction-Related Proteins in the Ischemic Cerebral Cortex

**Figure 9:**
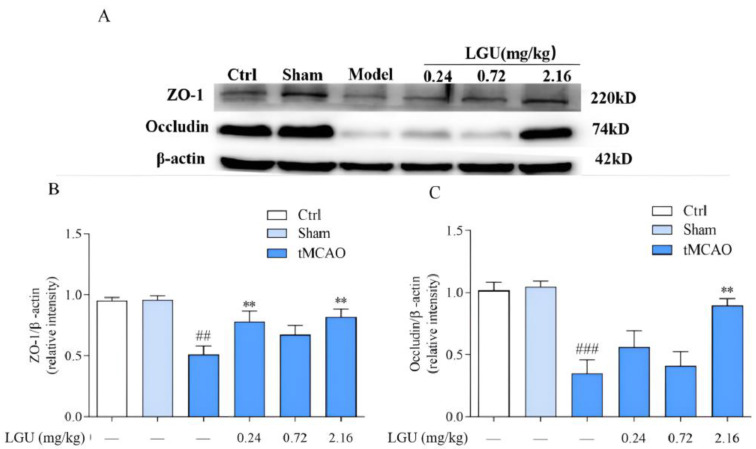
Effects of LGU on the expression of tight junction-related proteins in the ischemic cerebral cortex. (**A**): The expression of ZO-1 and Occludin of tignt junction proteins was examined by western blot analysis. (**B**): The relative expression of ZO-1 was normalized to β-actin. (**C**): The relative expression of Occludin was normalized to β-actin. Total protein was extracted from the ischemic cortex at 22 h of reperfusion after 2 h MCAO. n = 3. ## *p* < 0.01, ### *p* < 0.001, compared with the sham group; ** *p* < 0.01, compared with the model group.

The expression of tight junction-related proteins is shown in Figure 9. There was no significant change in ZO-1 or occludin between the control group and the sham operation group. After 24 h of ischemia/reperfusion, the tight junction proteins ZO-1 and occludin in the ischemic cerebral cortex of the model group were significantly decreased. A single tail vein injection of LGU significantly reduced the down-regulation of ZO-1 and occludin in brain tissue.

### 3.10. Effects of LGU on the Expression of Matrix Metalloproteinase in the Ischemic Cerebral Cortex

**Figure 10:**
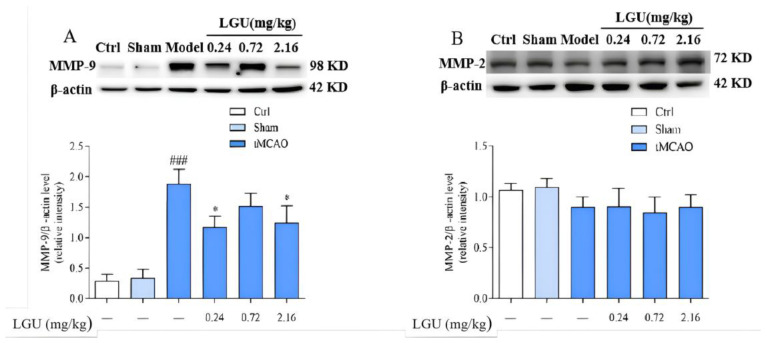
Effects of LGU on the expression of matrix metalloproteinase in the ischemic cerebral cortex. Expression levels of target proteins were detected by western blotting. (**A**): The expression of MMP-9 was examined by western blot analysis. (**B**): The expression of MMP-2 was examined by western blot analysis. Total protein was extracted from the ischemic cortex at 22 h of reperfusion after 2 h of MCAO. n = 3. ### *p* < 0.001, compared with the sham group; * *p* < 0.05, compared with the model group.

The expression of matrix metalloproteinase is shown in Figure 10. There was no significant difference between the control group and the sham operation group. Compared with the sham group, 24 h of ischemia/reperfusion did not affect the expression of MMP-2 in the ischemic cerebral cortex of the model group but significantly increased the expression of MMP-9. A single tail vein injection of LGU significantly reduced the up-regulation of MMP-9 protein expression in the ischemic cerebral cortex.

### 3.11. Effects of LGU on the Content of TNF-α and IL-1β in the Ischemic Cerebral Cortex of Rats

**Figure 11:**
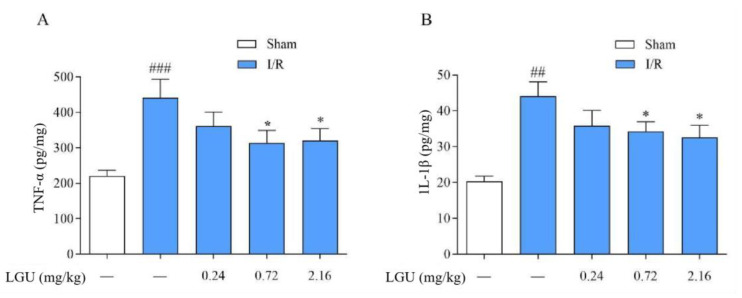
Effects of LGU on the content of TNF-α and IL-1β in the ischemic cerebral cortex of rats. (**A**): Effects of LGU on the content of TNF-α in the ischemic cerebral cortex of rats. (**B**): Effects of LGU on the content of IL-1β in the ischemic cerebral cortex of rats. The effects of LGU on tissue homogenate expression of TNF-α and IL-1β (pg/mg) at 22 h of reperfusion after 2 h of MCAO. Data are expressed as the mean ± S.E.M. n = 7. ## *p* < 0.01, ### *p* < 0.001, compared with the sham group; * p < 0.05, compared with the model group.

As shown in Figure 11, compared with the sham group, the contents of TNF-α and IL-1β in the ischemic cerebral cortex of the model group were significantly increased, while a single tail vein administration of LGU significantly reduced the contents of TNF-α and IL-1β in the ischemic cerebral cortex.

## 4. Discussion

In the present study, we observed that LGU effectively improved the decreased cell viability resulting from OGD/R and that LGU exhibited significant protective effects in the tMCAO rat model. Furthermore, Western blot analysis revealed that LGU administration via a single tail injection significantly reduced the expression levels of ZO-1 and occludin in brain tissue while also contributing to a significant reduction in the up-regulation of MMP-9 expression. Lastly, we observed that LGU administration effectively lowered the content of TNF-a and IL-1β in the ischemic cerebral cortex.

Cerebral ischemia/reperfusion injury refers to a series of rapid cascade reactions caused by the interruption of cerebral blood flow in brain tissue and the recovery of blood supply in brain tissue, which damage brain cells [24,25]. Its physiological and pathological mechanism is complex, including excitatory amino acid toxicity and energy disorder [26,27], free radical generation, inflammatory reactions [28,29], intracellular Ca ^2+^ overload, apoptosis, and other links, which interact and influence each other [5]. Studies have shown that one of the main risk factors for death in patients with early cerebral ischemia is increased BBB permeability [30]. In the early stage of cerebral ischemia/reperfusion injury, the BBB changes in both morphology and function, resulting in increased BBB permeability and increased brain edema, and the occurrence of brain edema further aggravates ischemic and hypoxic brain tissue damage [31,32]. Therefore, early intervention of BBB injury is one of the possible effective treatment measures to improve clinical prognosis.

The BBB is the core structure of the neurovascular unit. The dry–wet weight ratio of brain tissue, the content of Evans blue in brain tissue, and the expression of S100B in serum are important indicators that directly reflect the changes in BBB permeability [33]. As an azo dye, EB can be highly combined with plasma proteins in vivo [34]. Under normal physiological conditions, it cannot pass through the BBB. Only when the BBB of the nervous system is damaged, can EB enter the brain parenchyma through the damaged blood–brain barrier and make it blue [35]. Therefore, it is often used as a tracer to quantitatively determine the content of EB in brain tissue to observe the degree of BBB damage, which is a sensitive and simple method for the early detection of BBB damage [36]. Moreover, studies have shown that the degree of BBB damage is positively correlated with the EB content in the brain [37]. As a brain tissue-specific protein, the S100B protein is usually expressed in the cytoplasm and processes of astrocytes. In the case of the normal blood–brain barrier, it only appears in brain tissue and cerebrospinal fluid, and the content in the blood is very low. When the permeability of the blood–brain barrier increases, the S100B protein can enter the blood through the damaged blood–brain barrier, thereby increasing the content of the S100B protein in the blood. Therefore, monitoring the level of the S100B protein in the peripheral blood after craniocerebral injury can be used as one of the indicators to determine the degree of brain injury and prognosis [38]. The results of this study confirmed that BBB permeability increased after cerebral ischemia/reperfusion. Compared with the sham operation group, EB content in the ischemic brain tissue of rats in the model group was significantly increased, and EB permeability in the ischemic brain tissue of rats was significantly decreased after a single administration of LGU (0.72, 2.16 mg/kg) in the tail vein, with statistical significance. At the same time, compared with the sham operation, the serum S100B content of rats in the model group was significantly increased, and the serum S100B content of rats in the LGU (2.16 mg/kg) group was significantly decreased, indicating that LGU can reduce the degree of BBB destruction after cerebral ischemia/reperfusion. The ultrastructure of neurons and microvessels with a diameter <50 µm in brain tissue can be accurately observed and measured by transmission electron microscopy, especially the lumen of capillaries and vascular endothelial cells. After a single administration of LGU through the tail vein, each administration group could alleviate the structural discontinuity of the nuclear membrane and the uneven distribution of chromatin in the nucleus and improve the tight junction structure and basement membrane integrity.

The capillary endothelial cells and the tight junction structures (TJs) between them are the first barrier of the BBB and also an important structure to maintain the relative stability of the internal environment of brain tissue. TJs are the core structure of the BBB, which is composed of transmembrane proteins (claduin, occludin), junctional adhesion molecules (JAMs), cytoplasmic adhesion proteins (ZO-1, ZO-2, ZO-3), and cytoskeleton proteins [39]. The cytoplasmic adhesion protein ZO-1 and the transmembrane protein occludin are important components of endothelial cell TJs and play an important role in regulating BBB permeability. Occludin is the first isolated membrane protein with complete tight junctions. It is less expressed in endothelial cells outside the nervous system and is highly expressed in tight junctions in cerebrovascular endothelial cells. It is mainly involved in the formation of TJs in the nervous system, which is an important reason why the permeability of the blood–brain barrier is lower than that of other blood tissue barriers [40,41]. ZO-1 is an important structure in the connection between occludin and the intracellular skeleton system and signal transduction mechanism. It is highly expressed at the junction between endothelial cells, thereby maintaining the integrity of TJs [42]. One study found that in the process of stroke, with the prolongation of hypoxia time, the ZO-1 protein gradually migrated from the cell membrane to the cytoplasm and entered the nucleus, resulting in the breakage of the occludin–ZO-1 skeleton protein connection, resulting in increased blood–brain barrier permeability [43]. The expression of tight junction proteins ZO-1 and occludin, matrix metalloproteinase MMP-2, and MMP-9 in cerebral cortex tissue of cerebral ischemia/reperfusion rats at 24 h were quantitatively detected by the Western blot technique. The results showed that compared with the sham operation group, the tight junction proteins ZO-1 and occludin significantly decreased, the content of matrix metalloproteinase MMP-2 remained unchanged, and the content of MMP-9 significantly increased in the cerebral cortex tissue of cerebral ischemia/reperfusion rats. However, a single dose of LGU (0.24, 2.16 mg/kg) in the tail vein can up-regulate the expression of tight junction proteins ZO-1 and occludin and inhibit the expression of MMP-9 after cerebral ischemia/reperfusion, suggesting that LGU may up-regulate tight junction proteins ZO-1 and occludin. Inhibiting the expression of MMP-9 can restore the integrity of TJs, reduce the permeability of the BBB, and improve the degree of cerebral edema after cerebral ischemia/reperfusion.

Maintaining the integrity of the BBB function depends not only on vascular endothelial cells but also on the integrity of the capillary basement membrane and extracellular basement membrane. The latter two, also known as the ECM, have the functions of support, defense, and connection and are important components of the BBB [44]. MMPs are a class of highly conserved proteases in the evolution of nature. They belong to the zinc-and calcium-dependent endopeptidase family and can degrade all major components of the ECM, including collagen, gelatin, fibrin, and adhesion proteins. Under normal physiological conditions, the expression level of MMPs is extremely low, while under inflammatory conditions, such as cerebral ischemia/reperfusion, the expression level of MMPs is significantly up-regulated, which further leads to ECM degradation and BBB basement membrane rupture, increased BBB permeability, and brain edema. In the MMPS family, MMP-2 and MMP-9 are most closely related to blood–brain barrier injury. Studies have shown that in the early stage of ischemic stroke, the activity of MMP-2 was not significantly up-regulated. The expression of the MMP-2 protein increased at 120 h of cerebral ischemia/reperfusion in rats, while the activation of MMP-9 expression increased significantly at 4 h of reperfusion, peaked at 24 h of reperfusion, and returned to normal at 120 h of reperfusion [45]. The results of this experiment showed that compared with the sham group, after 24 h ischemia/reperfusion, there was no significant change in MMP-2 in the cerebral cortex of the ischemic side of rats in the model group, and MMP-9 was significantly increased. This is consistent with other reports in the literature. A single tail vein injection of LGU could significantly reduce the up-regulation of MMP-9 protein, which suggests that LGU can protect the BBB by reducing MMP-9.

IL-1β and TNF-α inflammatory mediators are the initiating factors of the inflammatory response. TNF-α is a cytokine with a wide range of biological functions, and it is also an important inflammatory factor in organisms, participating in the body’s immune response and inflammatory response [46]. Some scholars believe that TNF-α is directly related to BBB damage in cerebral ischemia/reperfusion injury and that TNF-α can directly activate white blood cells, increase the adhesion between white blood cells and endothelial cells, resulting in vascular hypoperfusion, and thus penetrate into the brain through the tight connection between endothelial cells and through the BBB to form cerebral edema. At the same time, the secretion of vasoactive substances is induced, which aggravates BBB injury [47]. TNF-α can also damage endothelial cells, up-regulate MMP protein expression, and further destroy BBB permeability [48]. IL-1β stimulates inflammation mainly by promoting the adhesion of white blood cells and endothelial cells. In the early stage of ischemic brain injury disease, cerebral ischemia/reperfusion injury induces macrophages to secrete IL-1β and TNF-α, activate white blood cells, and generate inflammatory response [49]. The Elisa results showed that compared with the sham operation group, the contents of IL-1β and TNF-α in the ischemic-side brain tissue of the model group were significantly increased. A single dose of LGU (0.72, 2.16 mg/kg) could significantly reduce the contents of TNF-α and IL-1β in the infarction-side brain tissue of the cerebral ischemia/reperfusion injury model. These results suggest that LGU can reduce inflammatory damage during cerebral ischemia/reperfusion injury by reducing inflammatory cytokines and thus play a protective role against cerebral ischemia.

Based on the experimental results, the findings suggest a promising clinical application for an improvement in neurological deficits following cerebral ischemia. Additionally, the reduction in brain edema and amelioration of pathological damage within the cerebral infarction zone suggest potential therapeutic advantages for individuals affected by these conditions. These results hold significant implications for the development of novel treatment approaches aimed at ameliorating the impact of cerebral ischemia and improving patient outcomes in clinical practice.

Recent studies have highlighted the critical role of increased intracranial pressure (ICP) and changes in intracranial compliance (ICC) in OGD/R [50]. Future research can enhance our results by exploring the connection between our findings and ICP/ICC changes, which are crucial for neuroclinician understanding. This investigation could provide valuable insights into the mechanisms underlying our observed effects and aid in the development of more effective treatment strategies for cerebral conditions.

## 5. Conclusions

In the present study, we demonstrated that LGU has a significant protective effect on cerebral ischemia/reperfusion injury in rats both in vitro and in vivo. It improved the cerebral nerve function score defect after cerebral ischemia/reperfusion, reduced the infarct volume ratio, alleviated cerebral edema, improved the pathological injury degree of cerebral infarction area, up-regulated the expression of tight protein ZO-1 and occludin, decreased the expression of matrix metal proteinase MMP-9 in the cerebral infarction area after cerebral ischemia/reperfusion, and reduced the inflammatory response.

## Data Availability

The data that support the findings of this study are available from the corresponding author upon reasonable request.
